# Effects of Diets Differing in Composition of 18-C Fatty Acids on Adipose Tissue Thermogenic Gene Expression in Mice Fed High-Fat Diets

**DOI:** 10.3390/nu10020256

**Published:** 2018-02-23

**Authors:** Sunhye Shin, Kolapo M. Ajuwon

**Affiliations:** 1 Interdepartmental Nutrition Program, Purdue University, West Lafayette, IN 47907, USA; shin157@purdue.edu; 2 Department of Animal Sciences, Purdue University, West Lafayette, IN 47907, USA

**Keywords:** 18-carbon fatty acids, brown-like phenotype, beige adipocytes, beta-adrenergic stimulation

## Abstract

Dietary fatty acids play important roles in the regulation of fat accumulation or metabolic phenotype of adipocytes, either as brown or beige fat. However, a systematic comparison of effects of diets with different composition of 18-C fatty acids on browning/beiging phenotype has not been done. In this study, we compared the effects of different dietary fats, rich in specific 18-carbon fatty acids, on thermogenesis and lipid metabolism. Male C57BL/6 mice were fed a control diet containing 5.6% kcal fat from lard and 4.4% kcal fat from soybean oil (CON) or high-fat diets (HFD) containing 25% kcal from lard and 20% kcal fat from shea butter (stearic acid-rich fat; SHB), olive oil (oleic acid-rich oil; OO), safflower oil (linoleic acid-rich oil; SFO), or soybean oil (mixed oleic, linoleic, and α-linolenic acids; SBO) *ad libitum* for 12 weeks, with or without a terminal 4-h norepinephrine (NE) treatment. When compared to SHB, feeding OO, SFO, and SBO resulted in lower body weight gain. The OO fed group had the highest thermogenesis level, which resulted in lower body fat accumulation and improved glucose and lipid metabolism. Feeding SFO downregulated expression of lipid oxidation-related genes and upregulated expression of lipogenic genes, perhaps due to its high n-6:n-3 ratio. In general, HFD-feeding downregulated *Ucp1* expression in both subcutaneous and epididymal white adipose tissue, and suppressed NE-induced *Pgc1a* expression in brown adipose tissue. These results suggest that the position of double bonds in dietary fatty acids, as well as the quantity of dietary fat, may have a significant effect on the regulation of oxidative and thermogenic conditions in vivo.

## 1. Introduction

Obesity, which is a condition of excessive adipose tissue accumulation [1], increases the risk of metabolic disorders, such as type 2 diabetes, dyslipidemia, and cardiovascular diseases [2]. Consumption of high fat diet contributes to obesity development because fatty acids increase caloric density of diets, and dietary fatty acids and their metabolites modulate the transcription of lipogenic and lipolytic genes [3]. However, fatty acids differentially regulate metabolism and exert divergent effects on obesity occurrence. For example, as compared to monounsaturated (MUFA) or saturated fatty acids (SFA), polyunsaturated fatty acids (PUFA) are potent suppressors of transcription of lipogenic genes through peroxisome proliferator-activated receptors (PPAR) mediated mechanisms [4], as antagonists of liver X receptors (LXR) [5], or by downregulating the expression of the sterol regulatory element-binding proteins (SREBP) [6].

PUFAs are also able to increase energy expenditure by stimulating thermogenesis in classical brown adipocytes and beige adipocytes [7,8,9,10]. Brown adipocytes are known to dissipate energy through thermogenesis, whereas white adipocytes mostly store energy [11]. However, beige adipocytes that reside in white adipose tissue (WAT) depots act like brown adipocytes in response to cold exposure and β-adrenergic stimulation [12]. Several studies have been done on the metabolic effects of fish oil-derived fatty acids, eicosapentaenoic acid (EPA) and docosahexaenoic acid (DHA), on adipose tissue phenotype. Treatment with EPA promoted thermogenic capacity in mouse primary white and brown adipocytes [7,8], and dietary fish oil upregulated thermogenic markers in murine brown adipose tissue (BAT) [9,10] and WAT [10]. However, very few studies have been conducted to compare the effects 18-carbon fatty acids, the most abundant dietary fatty acids, on the regulation of brown/beige phenotype and thermogenesis. 

Therefore, this study was conducted to examine effects of diets rich in different 18-carbon fatty acids on adipose tissue thermogenic gene expression profile. To investigate effects of different isomers of 18-carbon fatty acids, diets rich in shea butter (high stearic acid; STA, 18:0), olive oil (high oleic acid; OLA, 18:1), safflower oil (high linoleic acid; LNA, 18:2), or soybean oil (mixed OLA, LNA, and α-linolenic acid; ALA, 18:3) were fed to mice. We also tested effect of acute β-adrenergic stimulation with norepinephrine to determine if interaction existed between dietary fat and β-adrenergic receptor activation on thermogenic gene expression in adipose tissue and overall metabolic phenotype.

## 2. Materials and Methods

### 2.1. Animals and Diets

All of the animal procedures were approved and carried out in accordance with the regulations of the Purdue Animal Care and Use Committee. Four-week-old male C57BL/6J mice were purchased from the Jackson Laboratory and were maintained on a chow diet for four days before being assigned to one of five diet groups; control diet containing 5.6% kcal fat from lard and 4.4% kcal fat from soybean oil (CON) or high-fat diets (HFD) containing 25% kcal from lard and 20% kcal fat from shea butter (SHB; STA rich), olive oil (OO; OLA rich), safflower oil (SFO; LNA rich), or soybean oil (SBO; mixture of OLA, LNA, and ALA). All diets, including the CON diet, were based on purified ingredients to keep the diet matrices across diets the same for easy comparison. In addition, the ratio of vegetable oil to lard was kept at approximately 1.25 in all diets for uniformity. Diets were made to contain oils from different sources, rather than a single source, so that these diets would be more akin to human diets, which typically have oils from different sources, as much as possible.

Mice (*n* = 8 per group) were fed the experimental diets *ad libitum* for 12 weeks. Table S1 shows the composition of the experimental diets. Mice were housed in environmentally controlled housing (temperature = 72 ± 2 °F; humidity = 35 ± 5%), and a 12 hour-light-dark cycle. Body weight was measured once a week, and food intake was measured two times a week. At the end of the experimental period, mice were injected with norepinephrine (NE; 2 mg/kg body weight) or phosphate-buffered saline (PBS; 2 mL/kg body weight) after an 8-h fast, and euthanized with CO_2_ asphyxiation 4 h after the injection. Serum was separated from blood after centrifugation at 2000× *g* for 10 min at 4 °C. Subcutaneous and epididymal white adipose tissue (WAT), brown adipose tissue (BAT), liver, hamstring muscle, and hypothalamus were removed, weighed, and snap-frozen in liquid nitrogen, and stored at −80 °C until subsequent analyses.

### 2.2. Fatty Acid Composition of Diets

Total lipids were extracted from powdered diets using modified Folch method [13]. Fatty acids in extracted lipid samples were trans-esterified to form fatty acid methyl esters (FAME) with sodium methoxide in methanol. FAMEs were extracted with hexane, and the extracts were analyzed by gas chromatography (Varian 3900 gas chromatography system with CP-8400 autosampler; Agilent, Santa Clara, CA, USA). Fatty acid composition of diets is shown in Table 1.

### 2.3. Indirect Calorimetry

After eight weeks of feeding the experimental diets, O_2_ consumption (VO_2_) and CO_2_ production (VCO_2_) of mice (*n* = 4 per group) were measured for three days using the Oxymax Open Circuit Indirect Calorimeter (Columbus Instrument, Columbus, OH, USA). The respiratory exchange ratio (RER) was calculated as VCO_2_ divided by VO_2_. Heat production was calculated as VO_2_* (3.815 + 1.232*RER). Weight-normalized heat production was calculated as heat production divided by metabolic body weight (Wt^0.75^).

### 2.4. Intraperitoneal Glucose Tolerance Test

After 10 weeks of the experiment, glucose was administered by intraperitoneal injection to the mice (*n* = 4 per group) at 1.5 g/kg body weight after 8-h fasting. Blood was collected from the tail vein before the injection (0), and 15, 30, 45, 60, 120 min thereafter. Blood glucose concentration was determined using the FreeStyle Lite^®^ blood glucose meter kit (Abbott, Chicago, IL, USA), and the area under the curve (AUC) was calculated.

### 2.5. Lipid, Glucose, and Insulin Concentrations

Serum, hepatic, and muscular TAG was measured using the Sigma Triglyceride Reagent (Sigma-Aldrich, St Louis, MO, USA). Serum non-esterified fatty acid (NEFA) and glucose concentrations were measured using Wako HR Series NEFA-HR (2) and Wako Autokit Glucose (Wako Diagnostics, Mountain View, CA, USA). Serum insulin concentration was measured using the Ultra-Sensitive Mouse Insulin ELISA Kit (Crystal Chem, Chicago, IL, USA). Total lipids from the liver and hamstring muscle were extracted using isopropanol. After centrifugation, TAG content of the supernatant determined with the Sigma Triglyceride Reagent (Sigma-Aldrich, St Louis, MO, USA).

### 2.6. Quantitative Real-Time PCR

Total RNA was extracted from BAT, subcutaneous WAT (sWAT), epididymal WAT (eWAT), liver, hamstring muscle, and hypothalamus using Trizol (Ambion, Austin, TX, USA), and reverse transcribed to cDNA using M-MLV reverse transcriptase (Promega, Madison, WI, USA). Quantitative real-time PCR was performed on a Bio-Rad iQ5^®^ system (Bio-Rad, Hercules, CA, USA) using RT^2^ SYBR Green Fluor qPCR Mastermix (Qiagen, Valencia, CA, USA). The mRNA levels of *Ucp1*, *Prdm16*, *Pgc1a*, *Tfam*, *Cpt1a*, *Cpt1b*, *Cpt2*, *Ppara*, *Ppard*, *Pparg*, *Npy*, *Agrp*, *Pomc*, *Lepr*, *Adrb1*, *Adrb2*, *Adrb*, *Gatm*, and *Serca2b* were quantified and normalized relative to 18S rRNA. Fold changes of gene expression were calculated by the ΔΔCt method. Specific primer sequences used are shown in Table S2.

### 2.7. Statistical Analysis

One-way or two-way analysis of variance (ANOVA) with Tukey’s post-hoc test was performed to determine significant differences among groups. Data are presented with the means ± SEM and analyzed using SAS 9.4 (SAS Institute Inc., Cary, NC, USA).

## 3. Results

### 3.1. Body Weight, Calorie Intake, Growth Efficiency, and Tissue Weight

Body weights were significantly different among groups after 12 weeks of feeding (*P* < 0.0001). When compared to CON, SHB, and SBO groups had higher body weight (37% and 23% higher, respectively). The SHB group had a higher calorie intake (*P* = 0.0119). The SHB and SBO groups had higher growth efficiency (*P* = 0.0011) than CON. Subcutaneous and epididyimal WAT (sWAT and eWAT, respectively) weights (g WAT weight/100 g body weight) were also different. The SHB and SBO groups had 4.41 and 4.12 times higher sWAT weight (*P* = 0.0081); and 2.75 and 2.30 times higher eWAT weight (*P* = 0.0010) than CON (Table 2).

### 3.2. Indirect Calorimetry

**Figure 1:**
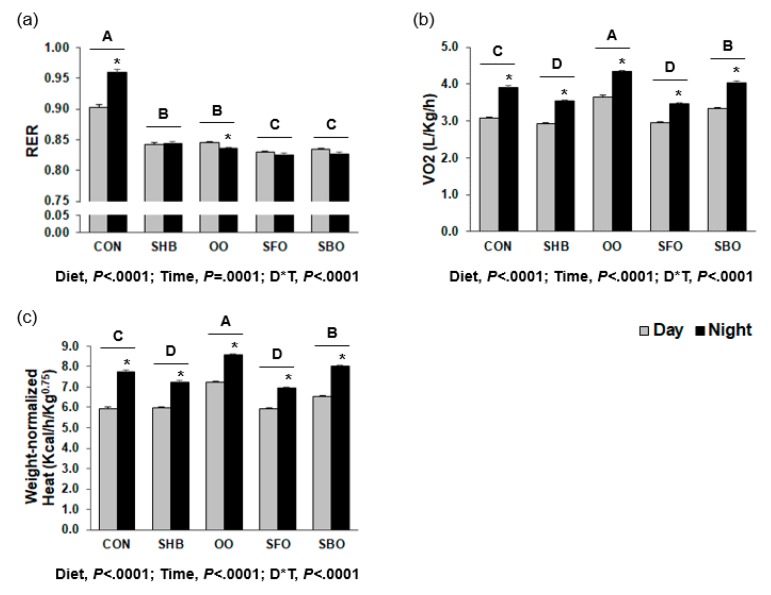
Indirect calorimetry (**a**) Respiratory exchange ratio (RER) (**b**) O_2_ consumption (**c**) Weight-normalized heat generation. CON, control; SHB, shea butter; OO, olive oil; SFO, safflower; SBO, soybean oil. After eight weeks of feeding the experimental diets, O_2_ consumption and CO_2_ production of mice were measured for three days using the Oxymax open circuit indirect calorimeter. Data were separated into day or night time for each diet. Bars represent means ± SEM (*n* = 4 for each diet group). Diets were compared among each other (after combining day and night data within each diet), and assigned different superscripts A, B, C, or D) if they were significantly different from each other at *P* < 0.05 by Tukey’s multiple comparison test. D*T, Interaction effect between diet and time. Asterisks (*) indicate significant differences due to time effect (day or night time) within each diet group when diet*time interaction effect was significant by ANOVA.

HFD-fed groups had lower respiratory exchange ratio (RER) than CON (*P* < 0.0001). As compared to CON, oxygen consumption and weight-normalized heat production were higher in the OO and SBO groups, and lower in the SHB and SFO groups (*P* < 0.0001; Figure 1).

### 3.3. Intraperitoneal Glucose Tolerance Test

**Figure 2:**
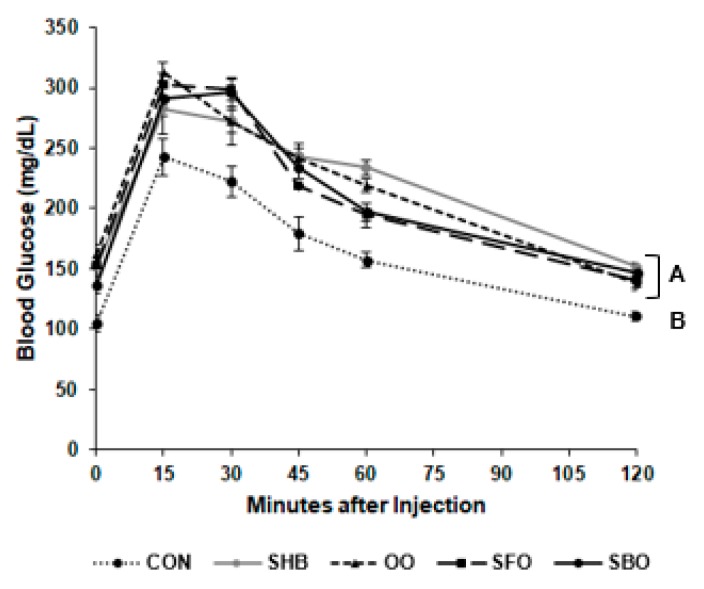
Blood glucose level during intraperitoneal glucose tolerance test. After 10 weeks of feeding the experimental diets, glucose was intraperitoneally injected to mice at 1.5 g/kg body weight after 8-h fasting. Blood was collected from the tail vein before the injection, and 15, 30, 45, 60, and 120 min after injection. Data are presented as means ± SEM (*n* = 4 for each group). Different superscript letters A and B indicate significant differences between all the high fat diets (shea butter (SHB), olive oil (OO), safflower oil (SFO), and soybean oil (SBO)), which all have a common superscript A, and the control (CON) diet with superscript B (*P* < 0.05) by Tukey’s multiple comparison test.

After glucose administration, blood glucose concentrations were higher in the HFD-fed groups when compared to CON (*P* < 0.0001; Figure 2). However, there was no difference in AUC among groups (*P* = 0.9257; data not shown).

### 3.4. Lipid, Glucose, and Insulin Concentrations

Serum and hepatic TAG concentrations were higher in the NE-injected than vehicle-treated mice (*P =* 0.0374, *P =* 0.0002, respectively). Compared to CON, the SFO group had a higher muscle TAG level (*P =* 0.0012). Serum NEFA concentrations were higher in NE-treated mice (*P =* 0.0044), and lower in SHB and OO groups than CON (*P =* 0.0146). Serum glucose and insulin concentrations were significantly higher in the SHB and SFO groups compared to CON (*P =* 0.0003, *P =* 0.0036, respectively), and NE-injected mice had lower serum glucose concentration than vehicle-treated mice (*P =* 0.0002). Glucose:insulin ratio was not different among groups (Table 3).

### 3.5. Protein Expression

Abundance of UCP1 protein was not different among groups in BAT and eWAT. Protein level of PGC1α was not different in BAT as well. The ratios of p-AKT/AKT, p-AMPK/AMPK, and p-PKA/PKA were not different among groups in BAT, eWAT, liver, and hamstring muscle (data not shown).

### 3.6. Regulation of Expression of Genes Involved in Thermogenesis in BAT

**Figure 3:**
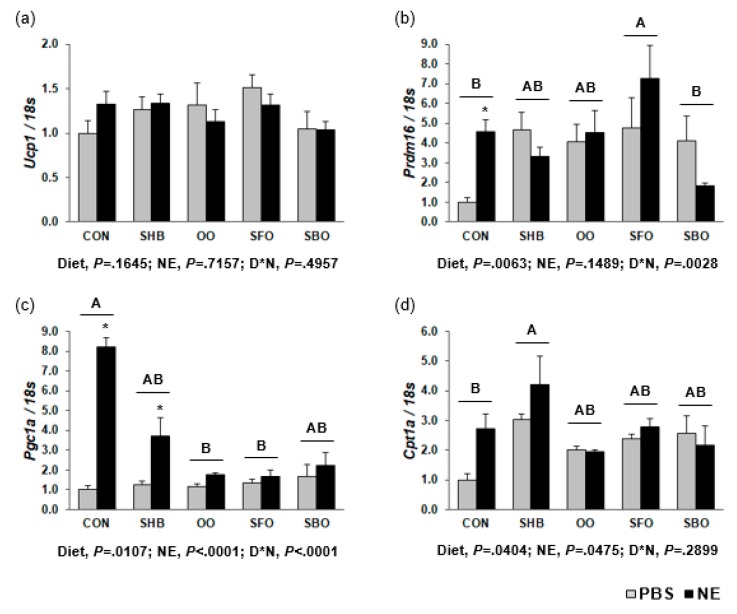
Expression of genes involved in thermogenesis in BAT (**a**) *Ucp1* (**b**) *Prdm16* (**c**) *Pgc1a* (**d**) *Cpt1a*. Mice were injected with norepinephrine (NE; 2 mg/kg body weight) or phosphate-buffered saline (PBS; 2 mL/kg body weight) as a vehicle after 8-h fasting at the end of the experimental period, and euthanized after another 4-h fasting. Gene expression was determined by RT-PCR. Bars represent means ± SEM (*n* = 8 for each diet group; *n* = 4 each for PBS and NE treatment). Diets were compared among each other (after combining PBS and NE data within each diet), and assigned different superscripts A, B, AB, C, or D) if they were significantly different from each other at *P* < 0.05 by Tukey’s multiple comparison test. If a diet has a common superscript with another diet, it means they are not significantly different from each other. Only diets without a common superscript are significantly different from each other. D*N, Interaction effect between diet and NE. Asterisks (*) indicate significant differences caused by NE within each diet group when interaction effect was significant. CON, control; SHB, shea butter; OO, olive oil; SFO, safflower; SBO, soybean oil.

Although there was no difference in *Ucp1* expression, *Prdm16* expression was higher in the SFO group as compared to CON (2.17-fold; *P =* 0.0063), and in CON, NE induced 4.55-fold higher *Prdm16* expression (*P =* 0.0002). When compared to CON, the OO and SFO groups had lower *Pgc1a* expression levels (0.32- and 0.33-fold, respectively; *P =* 0.0107), and NE upregulated its expression (*P* < 0.0001). The NE-induced *Pgc1a* upregulation was greater in the CON group than the HFD-fed groups. The level of *Cpt1a* mRNA expression was higher in the SHB group (1.95-fold; *P =* 0.0404), and NE treatment upregulated its expression (*P =* 0.0475; Figure 3). The SFO group had a higher *Adrb1* mRNA expression than the SBO group (*P =* 0.0158), and the SHB group had higher *Adrb3* mRNA expression than the OO group (*P =* 0.0571). Injection with NE downregulated *Adrb2* and *Adrb3* mRNA expression (*P =* 0.0028, *P =* 0.0187, respectively; Table S3).

### 3.7. Effect of Diets on Thermogenic Gene Expression in sWAT

**Figure 4:**
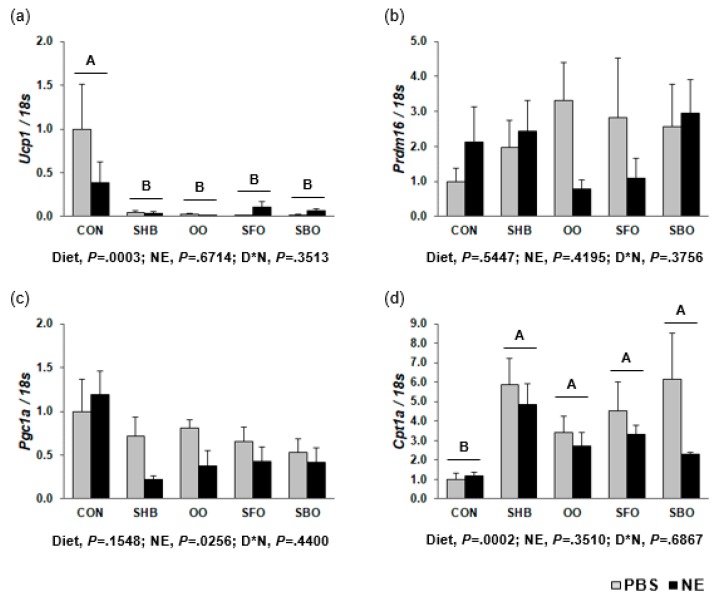
Expression of genes involved in thermogenesis in subcutaneous WAT (**a**) *Ucp1* (**b**) *Prdm16* (**c**) *Pgc1a* (**d**) *Cpt1a*. Mice were injected with norepinephrine (NE; 2 mg/kg body weight) or phosphate-buffered saline (PBS; 2 mL/kg body weight) as a vehicle after 8-h fasting at the end of the experimental period, and euthanized after another 4-h fasting. Gene expression was determined by RT-PCR. Data are presented as means ± SEM (*n* = 8 for each diet group; *n* = 4 each for PBS and NE). Diets were compared among each other (after combining PBS and NE data within each diet), and assigned different superscripts A or B) if they were significantly different from each other at *P* < 0.05 by Tukey’s multiple comparison test. If a diet has a common superscript with another diet, it means they are not significantly different from each other. Only diets without a common superscript are significantly different from each other. D*N, Interaction effect between diet and NE. CON, control; SHB, shea butter; OO, olive oil; SFO, safflower; SBO, soybean oil.

Expression of *Ucp1* in the HFD-fed groups was lower than the CON group (0.02- to 0.08-fold; *P =* 0.0003), and *Ucp1* expression level in sWAT was negatively correlated with sWAT percentage (r = −0.3117, *P =* 0.0502). There was no diet effect on *Pgc1a* mRNA expression, but NE lowered its level in the HFD-fed groups (*P =* 0.0256). Furthermore, the HFD-fed groups had higher *Cpt1a* mRNA level (*P =* 0.0002; Figure 4). When compared to CON, the SHB group had a higher *Tfam* mRNA level (*P =* 0.0037). The levels of β-adrenergic receptor mRNA were not different among the groups; however, NE downregulated *Adrb3* mRNA expression (*P =* 0.0012). The OO group had lower *Serca2b* mRNA expression than SHB group (*P =* 0.0281; Table S4). 

### 3.8. Effect of Diets on Thermogenic Gene Expression in eWAT

**Figure 5:**
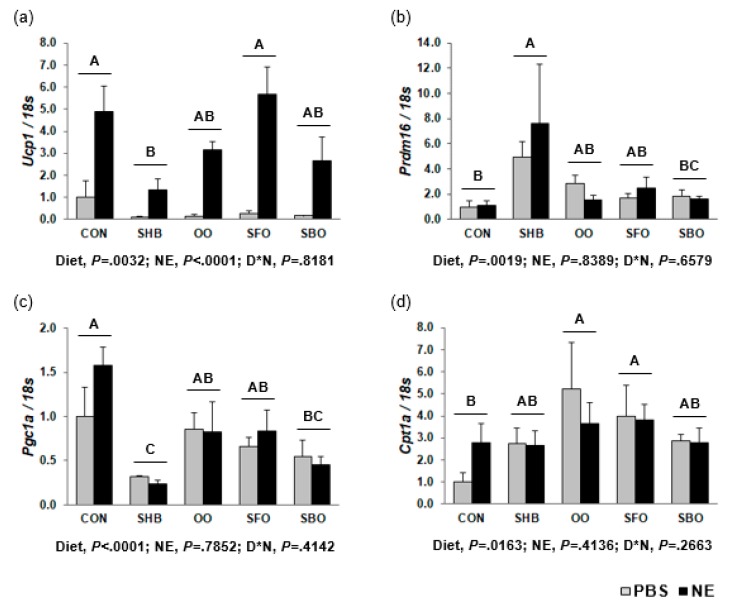
Expression of genes involved in thermogenesis in epididymal WAT (**a**) *Ucp1* (**b**) *Prdm16* (**c**) *Pgc1a* (**d**) *Cpt1a*. Mice were injected with norepinephrine (NE; 2 mg/kg body weight) or phosphate-buffered saline (PBS; 2 mL/kg body weight) as a vehicle after 8-h fasting at the end of the experimental period, and euthanized after another 4-h fasting. Gene expression was determined by RT-PCR. Data are presented as means ± SEM (*n* = 8 for each diet group; *n* = 4 each for PBS and NE). Diets were compared among each other (after combining PBS and NE data within each diet), and assigned different superscripts A, B, AB, BC, or C) if they were significantly different from each other at *P* < 0.05 by Tukey’s multiple comparison test. If a diet has a common superscript with another diet, it means they are not different. Only diets without a common superscript are significantly different. D*N, Interaction effect between diet and NE. CON, control; SHB, shea butter; OO, olive oil; SFO, safflower; SBO, soybean oil.

The SHB group had a lower *Ucp1* expression than CON (0.24-fold; *P =* 0.0032), and NE treatment upregulated *Ucp1* expression (*P* < 0.0001). Expression level of *Ucp1* in eWAT was negatively correlated with body weight increase (r = −0.3156, *P =* 0.0472). Expression of *Prdm16* was higher in the SHB group (5.91-fold; *P =* 0.0019). Additionally, the SHB and SBO groups had lower *Pgc1a* mRNA expression (0.21- and 0.39-fold, respectively; *P* < 0.0001), and *Pgc1a* expression level was negatively correlated with body weight increase (r = −0.7163, *P* < 0.0001) and eWAT percentage (r = −0.5306, *P =* 0.0004). When compared to CON, the OO and SFO groups had a higher *Cpt1a* mRNA level (2.35- and 2.10-fold, respectively; *P =* 0.0163; Figure 5), *Tfam* expression level (2.09- and 1.73-fold, respectively; *P =* 0.0012), and *Adrb1* mRNA level (1.92- and 3.83-fold; *P =* 0.0010). The SHB, OO, and SBO groups had lower *Adrb2* mRNA expression (0.11-, 0.35-, and 0.45-fold; *P* < 0.0001), and the SHB group had lower *Adrb3* mRNA expression (0.23-fold; *P =* 0.0115) than CON (Table S5).

### 3.9. Dietary Effects on Expression of Genes Involved in Lipid Metabolism in the Liver

Treatment with NE upregulated *Cpt1a* and *Cpt1b* expression (*P =* 0.0014, *P* < 0.0001, respectively). The SHB group had a higher *Cpt1a* expression (1.56-fold; *P* < 0.0001), and the SFO group had lower *Cpt2* expression (0.37-fold; *P* < 0.0001) than CON. As compared to CON, the SHB and OO groups had a higher *Ppara* expression (2.70- and 2.03-fold, respectively; *P* < 0.0001). Likewise, the SHB and SBO groups had a higher *Ppard* expression than CON (1.86- and 1.49-fold, respectively; *P* < 0.0001). The SHB, SFO, and SBO groups also had higher *Pparg* expression than CON (10.86-, 5.55-, and 9.15-fold, respectively; *P* < 0.0001). In addition, when compared to CON, the SFO group had a lower expression of *Adrb2* (0.49-fold; *P* < 0.0001), whereas the SHB, OO, and SBO groups had a higher *Adrb3* expression (2.74-, 3.01-, and 2.78-fold, respectively; *P* < 0.0001) relative to CON. However, treatment with NE downregulated *Adrb3* expression (*P =* 0.0060, Table S6).

### 3.10. Regulation of Expression of Genes Involved in Lipid Metabolism in Hamstring Muscle

When compared to CON, the SHB group had a higher *Cpt1a* expression (2.38-fold; *P =* 0.0056), and the HFD-fed groups had higher *Cpt1b* expression (1.62- to 3.04-fold; *P* < 0.0001). Expression of *Ppara* was higher in SHB, SFO, and SBO groups (3.07-, 2.28-, and 2.02-fold, respectively; *P =* 0.0008), and the SHB group had a higher *Pparg* expression (8.34-fold; *P =* 0.0010) than CON. Expression of *Ppard* was higher in SHB and OO groups (2.43- and 1.79-fold, respectively; *P =* 0.0003), and NE upregulated its expression (*P =* 0.0003). Expression of *Adrb1* was higher in SHB and SFO groups than CON (3.60- and 3.02-fold; *P =* 0.0062) (Table S7).

### 3.11. Expression of Neuropeptides and β-Adrenergic Receptors in Hypothalamus in Response to Diets

β-adrenergic receptor mRNA level was significantly different among the groups although *Npy*, *Agrp*, *Pomc*, and *Lepr* mRNA expression were not. As compared to the SBO group, the SFO group had lower *Adrb1* and *Adrb3* mRNA expression (*P =* 0.0369, *P =* 0.0074, respectively); and, the CON, OO, and SFO groups had lower *Adrb2* mRNA expression (*P =* 0.0001, Table S8).

## 4. Discussion

We have investigated the effects of dietary fatty acid isomers with 18 carbons on thermogenesis and metabolic markers in HFD-fed mice. Proportionately, 18-carbon fatty acids represent the largest fatty acid class in human diets, but their relative metabolic effects on thermogenic gene expression in adipose tissue has not been well studied. Overall effects of dietary fatty acids in energy balance is evident in the weight gain after consumption of the diets. Mice fed SHB (STA-rich fat) had the highest weight gain, growth efficiency, and white adipose mass. The SHB group also had the lowest oxygen consumption and heat production among the five dietary groups. It is possible that the downregulation of thermoregulatory genes, including *Ucp1*, *Pgc1a*, and *Adrb3* in eWAT of the SHB group is partly responsible for the higher weight gain in this group. SHB group had much higher serum glucose and insulin concentrations than CON, which may suggest an existence of insulin resistance in this group.

However, when compared to SHB-fed mice, mice fed OO (OLA-rich oil), SFO (LNA-rich oil), or SBO (mixture of OLA, LNA, and ALA) had lower body weight gain. Among HFD-fed groups, OO-fed mice had the least body fat accumulation. This may be tied to the highest oxygen consumption and heat production in this group. In support of this possibility, the OO group also had higher expression of epididymal thermoregulatory markers, *Pgc1a*, *Adrb2*, and *Adrb3* than SHB group, and higher *Tfam*, *Cpt1a*, and *Adrb1* than CON. This result will be in agreement with the previously reported thermogenic effects of OO [14,15]. Rats fed OO had higher expression levels of *Ucp1*, *Ucp2*, and *Ucp3* in BAT, and *Ucp3* in skeletal muscle, and this was associated with increased total body oxygen consumption [14]. Feeding OO also increased energy expenditure in healthy normal weight men [15], and the Mediterranean diet, which is rich in OO, was shown to significantly reduce body weight and body mass index in multiple randomized controlled trials [16]. Oleic acid, an n-9 fatty acid, potentially contributes to the thermogenic phenotype of the OO-fed mice. In C2C12 myocytes, OLA stimulates β-adrenergic signaling by increasing cyclic adenosine monophosphate (cAMP) concentration and protein kinase A (PKA) activity, and eventually induces PGC-1α activation and fatty acid oxidation [17]. In the current study, OO feeding tended to attenuate the increase in serum glucose and insulin concentrations caused by HFD feeding. This could reflect the metabolic effects of OLA in the OO diet in increasing fatty acid oxidation. This potential effect could result in increased clearance of excessive fatty acids and reduce obesity-induced chronic inflammation. 

It is quite interesting that oxygen consumption and heat production in SFO-fed mice were as low as SHB-fed mice even though SFO contains higher percentage of PUFA as compared to SHB. Serum glucose and insulin concentrations in the SFO group were also similar to the SHB group. This may indicate that dietary PUFA level alone may not be sufficient to determine metabolic outcome, and suggests a possibility that the balance of individual fatty acids in the diets may be critical. In this respect, the SFO diet has a high content of LNA. In the body, LNA may be elongated to arachidonic acid (ARA), which is a fatty acid that has been reported to inhibit brown-like phenotype acquisition of differentiated human multipotent adipose-derived stem cells [18], in contrast to n-3 fatty acids that upregulated thermogenic markers in WAT [10,19]. The high n-6:n-3 ratio in SFO could also be a possible link to the low thermogenesis in the SFO group. A link between a high n-6:n-3 ratio and insulin resistance has previously been reported [20].

However, mice fed the SBO diet, rich in LNA, had higher oxygen consumption and heat production than SFO-fed mice. This may be because SBO contains ALA, and the n-6:n-3 ratio of SBO is much lower than that of SFO. The SBO group had higher body weight, growth efficiency, and body fat accumulation. Previous studies showed that SBO increased feed efficiency and weight gain in mice [21], and dietary LNA increased body fat mass [22] and adipocyte size [22,23] in mice. It was also reported that LNA increased activity of endogenous lipid mediators synthesized from ARA in membrane phospholipids, and these induce obesity by upregulating de novo fatty acid synthesis in the liver [21]. Although ALA can be converted to EPA and DHA that are known prevent obesity and inflammation, the efficiency of this conversion is very low [24,25]. Therefore, the extra LNA in the diet of SBO fed mice could potentially contribute to the body fat accumulation in these mice.

We also determined possible combined effects of dietary fatty acids and β-adrenergic stimulation on thermogenesis. Fatty acids are known natural ligands for PPARs, such as PPARγ [4], which bind to peroxisome proliferator response element (PPRE) within the promoter region of genes such as PGC-1α. Fatty acids may also promote interaction of PPARγ with PGC-1α, leading to the induction of thermogenic genes, including *Ucp1* [12]. Stimulation of β-adrenergic signaling is a known mechanism of thermogenic activation in BAT and induction of oxidative genes in multiple tissues that may alter the oxidation and metabolism of the fatty acids provided in the diets [26]. To do this, we injected NE (a non-selective β-adrenergic receptor agonist) to mice 4 h before sacrifice. The observed elevation of serum TAG and NEFA and hepatic TAG concentration in NE-injected mice, and the lower serum glucose concentration compared to vehicle-injected mice, would be consistent with activation of beta-adrenergic stimulus. β-adrenergic stimulation induces lipolysis in adipose tissue and glucose uptake in skeletal muscle and adipose tissue [27]. These data indicate the activation of β-adrenergic signaling and a possible redirection of adipose TAG into the liver.

In BAT, NE upregulated expression of mitochondrial genes, such as *Pgc1a* and *Cpt1a*, and its effect was greater in the CON group than HFD-fed groups. Although NE treatment induced >8-fold higher *Pgc1a* expression in CON group, it only induced 1.5- to 3-fold higher expression in the HFD-fed groups. In addition, NE was effective in inducing *Cpt1a* expression in the CON group (2.72-fold), but its effect in the HFD-fed groups was slight and insignificant (average of 1.11-fold in all HFD-fed groups). Although there was no overall effect of NE on *Prdm16* expression, NE-injected CON mice had 4-fold higher expression compared to PBS-injected CON mice. Feeding HFD was previously reported to reverse adenylyl cyclase activation induced by epinephrine or CL316,243 treatment in BAT of C57BL/6 mice [28]. These data imply that high-fat feeding suppresses thermogenic response of BAT to β-adrenergic stimulation. These data may also indicate that HFD with different fatty acid composition similarly inhibited the NE effect; thus, fatty acid level, not composition, may be important in this respect.

The reported downregulation of *Adrb3* in BAT and in sWAT by NE has also been reported before. Administration of NE increased thyroid hormone secretion in mice [29], and thyroid hormone downregulated β_3_-AR expression at the mRNA level in BAT [30]. Downregulation of adrenergic receptor expression by NE is a known mechanism of adrenergic receptor desensitization [31], and this is a mechanism to prevent an overstimulation of the pathway. The lower expression of *Pgc1a* in sWAT of NE-treated mice could be related to the NE-induced downregulation of *Adrb3* in sWAT. Additionally, *Adrb3* gene transcript has been shown to be degraded by treatment of insulin [32] and glucocorticoids [33] in 3T3-F442A adipocytes, suggesting that *Adrb3* mRNA level is tightly regulated to control fat mobilization and oxidation [34]. The reduction in the *Adrb2* expression in BAT also appears related to the general theme of adrenergic receptor downregulation and desensitization by prolonged NE exposure.

Administration of NE led to upregulated *Ucp1* expression in eWAT but not sWAT. Subcutaneous fat depots are known to have more beige precursor cells than visceral fat depots [35]. Consistent with this observation, we also observed much higher expression of *Ucp1* in sWAT as compared to eWAT (Figure S1). Chronic activation of β-adrenergic signaling is known to induce trans-differentiation of pre-existing beige adipocytes; therefore, subcutaneous fat depots acquire brown-like phenotype more than visceral fat depots [36]. However, CL316,243, a β_3_-AR agonist, was reported to increase the number of BrdU+ UCP1+ cells in epididymal fat but not in the inguinal fat [37]. This indicates that β-adrenergic stimulation initiates proliferation of beige precursor cells in visceral fat depots, prior to inducing trans-differentiation, and this is potentially for robust induction of brown-like phenotype in the browning-resistant fat depots [36]. This mechanism may be connected to the observed upregulation of *Ucp1* by NE in eWAT in this study.

Feeding of HFD suppressed acquisition of brown-like phenotype in both sWAT and eWAT by downregulating *Ucp1* expression. We also observed a negative correlation between body adiposity (percentage of sWAT and eWAT, and body weight increase) and *Ucp1* expression level in sWAT and eWAT. This indicates that the *Ucp1* expression level might be important in determining the overall degree of body adiposity, perhaps by setting the threshold of wasteful energy utilization. In support of this possibility, lower *Ucp1* expression was previously observed in subcutaneous fat of HFD-fed obese rats [38] and in differentiated beige adipocytes derived from human subcutaneous WAT of obese individuals [39]. In mice, high-fat feeding induced differentiation of beige precursor cells in epididymal fat toward to white adipocytes [37]. Obesity has also been shown to inhibit metabolic responses of BAT to ephedrine [40], insulin, or cold exposure [41]; however, it is still unknown whether HFD-induced *Ucp1* downregulation is related to causation, or whether it is a consequence of obesity [39]. We also observed that HFD feeding downregulated expression of *Adrb2* and *Adrb3* in eWAT. Similar results have been reported in obese individuals [42]. In both visceral and subcutaneous fat, expression of *Adrb2* and *Adrb3* was lower in the obese when compared to normal weight individuals [42]. Due to the major role of β_2_- and β_3_-AR in activating lipolysis to provide fatty acids for thermogenesis [43], lower expression of *Adrb2* and *Adrb3* may be linked to the higher body fat accumulation in HFD-fed groups.

To determine if UCP1-independent thermogenic mechanisms were also involved in the observed metabolic phenotype from diets and NE injection, we measured the expression of *Gatm* [44] and *Serca2b* [45], which were recently reported to enhance UCP1-independent thermogenesis in beige fat. However, expression of *Gatm* mRNA was not different among groups in both sWAT and eWAT, *Serca2b* expression in sWAT was only lower in the OO group than SHB group. The lack of a major treatment effects on these genes suggest that these UCP1-independent thermogenic mechanisms might not have played a significant role in the metabolic phenotype that was observed in this experiment.

Although our major focus was the effect of diets and NE on adipose tissue metabolic markers, we also investigated effects on the liver and skeletal muscle. Hepatic TAG levels were higher in SHB, SFO, and SBO groups than CON group, although this was not statistically significant. When compared to CON group, the SHB group had 11-fold higher hepatic *Pparg* mRNA expression; however, fatty acid oxidation-related genes, such as *Cpt1a*, *Ppara*, and *Ppard*, were also upregulated in this group. It has been previously reported that patients suffering from non-alcoholic fatty liver disease (NAFLD) [46] and obese individuals with insulin resistance [47] had greater hepatic mitochondrial oxidation, indicating the SHB diet may be making more fatty acids available for both hepatic storage and oxidation. However, as compared to the SHB group, the OO group had lower *Pparg* expression in the liver, as previously reported that OO-rich Mediterranean diet reduced intrahepatic lipid level even without weight reduction in NAFLD patients [48]. Downregulation of *Cpt1a* and *Cpt2* expression in OO group may be caused by the lower level of liver fat available for oxidation. On the other hand, the SFO-fed mice had lower *Adrb1*, *Adrb2*, *Adrb3*, *Cpt1a*, *Cpt2*, *Ppara*, and *Ppard* expression than OO-fed mice despite their higher *Pparg* expression. NAFLD patients are known to have higher n-6: n-3 ratio in hepatic phospholipids [49]; therefore, the high n-6:n-3 ratio of SFO could have led to an induction of lipogenesis and the suppression of lipid oxidation or secretion in the liver.

Results obtained in the muscle mirrors those of the liver. SHB-fed mice had 8-fold higher *Pparg* expression in the hamstring muscle as compared to CON group. They also had higher mRNA levels of *Cpt1a*, *Cpt1b*, *Ppara*, and *Ppard*, perhaps indicating a higher level of both fatty acid storage and oxidation in the skeletal muscle and liver of the SHB-fed mice. However, consumption of n-6 fatty acid rich diets leads to downregulation of muscle oxidative genes in the present study. When compared to SHB-fed mice, SFO-fed mice had lower *Cpt2* and *Ppard* expression, leading to higher muscle TAG level; and, SBO-fed mice also had lower *Cpt1a*, *Ppard*, and *Adrb1* expression. Omega-6 fatty acids were previously observed to induce higher muscle TAG accumulation in rats [50]. Treatment with NE seemed to promote fatty acid oxidation by upregulating *Ppara* and *Ppard* expression and downregulating *Pparg* expression. Downregulation of *Adrb3* mRNA level by NE implies that muscular fat oxidation is also tightly controlled by negative feedback regulation through adrenergic receptor downregulation [34].

Finally, we determined the effects of diets on hypothalamic expression of neuropeptides, leptin receptor, and β-adrenergic receptors because overall metabolism and thermogenesis is under hypothalamic control. Although neuropeptide Y (NPY; an orexigenic neuropeptide) is known to suppress thermogenesis of BAT and browning of WAT [51], hypothalamic expression of neuropeptides, including NPY, was not different among groups. However, SFO group had the lowest hypothalamic *Adrb2* expression, and this could be caused by the high n-6: n-3 ratio in SFO. In support of this possibility, β-adrenergic receptor expression is upregulated by n-3 rich fish oil in murine brains [52], but downregulated by n-3 deficient diet in rat hypothalamus [53]. Central β-agonist treatment is also reported to induce thermogenesis by increasing BAT mass and browning of WAT [54]. Therefore, the low hypothalamic *Adrb2* expression in the SFO group could contribute the lower oxygen consumption and heat generation in this group.

## 5. Conclusions

In sum, dietary OO consumption reduced weight gain and body fat accumulation, and this is associated with upregulation of thermogenesis, and improved lipid metabolism in the liver and skeletal muscle. The high n-6:n-3 ratio of SFO increased lipogenesis and reduced lipid oxidation and thermogenesis. However, overall HFD feeding led to the suppression of acquisition of brown-like phenotype in both sWAT and eWAT, and the responsiveness of BAT to NE treatment. This might be due to the HFD-induced downregulation of *Adrb2* and *Adrb3* in sWAT and eWAT, and the lower expression of *Ucp1* in these fat tissues. These data indicate that both quantity and quality of dietary fat, especially the position of double bonds in fatty acids, may play key roles in the regulation of thermogenic response in diets.

## Figures and Tables

**Table 1:** Fatty acid composition of the experimental diets (% of fatty acids) ^1^.

	10% Fat	45% Fat			
	CON	SHB	OO	SFO	SBO
Myristic acid (C14:0)	1.046	0.728	0.681	0.709	0.685
Palmitic acid (C16:0)	17.148	13.287	18.595	13.887	15.740
Palmitoleic acid (C16:1, ∆9)	0.976	0.998	1.863	0.963	0.944
Stearic acid (C18:0)	8.604	23.781	7.503	7.144	7.808
Oleic acid (C18:1, ∆9)	27.793	38.828	44.971	27.233	28.916
Linoleic acid (C18:2, ∆9,12)	32.187	15.201	18.772	43.238	35.580
α-Linolenic acid (C18:3, ∆9,12,15)	3.374	0.858	1.023	0.817	3.731
Arachidonic acid (C20:4, ∆5,8,11,14)	0.142	0.159	0.148	0.149	0.154
Eicosapentaenoic acid (C20:5, ∆5,8,11,14,17)	0.100	0.011	0.022	0.066	0.032
Docosahexaenoic acid (C22:6, ∆4,7,10,13,16,19)	0.072	0.044	0.037	0.060	0.062
Total saturated fatty acids	27.552	38.422	27.498	22.465	24.986
Total monounsaturated fatty acids	30.803	41.411	49.470	29.937	31.784
Total polyunsaturated fatty acids	37.663	18.221	21.519	45.818	41.364
Total n-6 fatty acids	32.976	16.031	19.549	44.059	36.412
Total n-3 fatty acids	3.822	1.622	1.397	1.213	4.097
n-6:n-3 ratio	8.628	9.883	13.994	36.322	8.887

CON, 4.4% soybean oil + 5.6% lard; SHB, 20% shea butter + 25% lard; OO, 20% olive oil + 25% lard; SFO, 20% safflower oil + 25% lard; SBO, 20% soybean oil + 25% lard. ^1^ Fatty acid composition was determined by a gas chromatography.

**Table 2:** Body weight, calorie intake, growth efficiency, and tissue weight.

	10% Fat	45% Fat				*P*-Value
	CON	SHB	OO	SFO	SBO	
Body weight at 0 week (g)	17.88 ± 0.61	17.73 ± 0.51	17.83 ± 0.50	17.89 ± 0.45	17.88 ± 0.62	0.9931
Body weight at 12 week (g)	26.00 ± 0.69 ^C^	35.79 ± 0.81 ^A^	29.33 ± 0.66 ^BC^	30.41 ± 0.90 ^BC^	31.95 ± 1.93 ^AB^	<0.0001
Body weight gain (g)	8.13 ± 0.59 ^C^	18.06 ± 0.71 ^A^	11.50 ± 0.73 ^BC^	12.33 ± 0.93 ^B^	14.08 ± 1.47 ^B^	<0.0001
Calorie intake (kcal/day)	9.27 ± 0.28 ^B^	12.97 ± 0.02 ^A^	10.68 ± 0.09 ^AB^	10.43 ± 0.19 ^B^	11.14 ± 0.04 ^AB^	0.0119
Growth efficiency (mg/kcal) ^1^	10.39 ± 0.57 ^B^	16.58 ± 0.65 ^A^	12.82 ± 0.82 ^AB^	14.01 ± 0.89 ^AB^	15.03 ± 1.57 ^A^	0.0011
Subcutaneous WAT (g/100 g body weight)	0.59 ± 0.09 ^B^	2.60 ± 0.46 ^A^	1.25 ± 0.17 ^AB^	1.88 ± 0.45 ^AB^	2.43 ± 0.64 ^A^	0.0081
Epidydimal WAT (g/100 g body weight)	1.38 ± 0.18 ^C^	3.80 ± 0.42 ^A^	2.12 ± 0.21 ^BC^	2.79 ± 0.51 ^ABC^	3.18 ± 0.48 ^AB^	0.0010
Brown adipose tissue (g/100 g body weight)	0.34 ± 0.02 ^AB^	0.38 ± 0.04 ^A^	0.20 ± 0.02 ^C^	0.24 ± 0.03 ^BC^	0.36 ± 0.05 ^AB^	0.0009
Liver (g/100 g body weight)	3.72 ± 0.18	3.36 ± 0.12	3.33 ± 0.06	3.22 ± 0.11	3.21 ± 0.19	0.0956
Thigh (g/100 g body weight)	0.86 ± 0.04	0.80 ± 0.05	1.05 ± 0.06	0.95 ± 0.08	1.06 ± 0.14	0.1160

Data are presented as means ± SEM, *n* = 8 for each group. CON, 4.4% soybean oil + 5.6% lard; SHB, 20% shea butter + 25% lard; OO, 20% olive oil + 25% lard; SFO, 20% safflower oil + 25% lard; SBO, 20% soybean oil + 25% lard. For each variable, diets were compared among each other and means assigned different superscripts A, AB, BC, or C) if they are significantly different from each other at *P* < 0.05 by Tukey’s multiple comparison test. For any variable, if a diet has a common superscript with another diet, it means they are not significantly different from each other. Only diets without a common superscript are significantly different from each other. ^1^ Growth efficiency (mg/kcal) = Weight gain (mg)/Total calorie intake (kcal).

**Table 3:** Lipid, Glucose, and Insulin Concentrations.

	TRT ^1^	10% Fat	45% Fat				TRT Mean ^3^	*P*-Value ^4^
		CON	SHB	OO	SFO	SBO		
Serum TAG (mg/mL)	PBS	0.87 ± 0.05	0.69 ± 0.08	0.98 ± 0.20	1.00 ± 0.12	0.85 ± 0.09	0.87 ± 0.05	D; 0.9803
	NE	1.01 ± 0.17	1.21 ± 0.08	0.97 ± 0.20	0.98 ± 0.10	1.07 ± 0.12	1.05 ± 0.06 *	N; 0.0374
	Diet mean ^2^	0.94 ± 0.09	0.95 ± 0.11	0.97 ± 0.13	0.99 ± 0.07	0.96 ± 0.08		D*N; 0.1894
Liver TAG (mg/mg protein)	PBS	0.19 ± 0.04	0.22 ± 0.01	0.17 ± 0.04	0.21 ± 0.05	0.21 ± 0.07	0.20 ± 0.02	D; 0.5666
	NE	0.28 ± 0.04	0.36 ± 0.03	0.30 ± 0.01	0.35 ± 0.05	0.39 ± 0.10	0.34 ± 0.02 *	N; 0.0002
	Diet mean ^2^	0.23 ± 0.03	0.29 ± 0.03	0.24 ± 0.03	0.28 ± 0.04	0.30 ± 0.07		D*N; 0.9033
Muscle TAG (mg/mg protein)	PBS	0.21 ± 0.03	0.16 ± 0.02	0.20 ± 0.02	0.24 ± 0.03	0.22 ± 0.02	0.20 ± 0.01	D; 0.0012
	NE	0.16 ± 0.01	0.16 ± 0.02	0.24 ± 0.02	0.34 ± 0.06	0.19 ± 0.03	0.22 ± 0.02	N; 0.8868
	Diet mean ^2^	0.18 ± 0.02 ^B^	0.16 ± 0.01 ^B^	0.22 ± 0.02 ^AB^	0.30 ± 0.04 ^A^	0.20 ± 0.02 ^AB^		D*N; 0.1315
Serum NEFA (mM)	PBS	0.077 ± 0.003	0.058 ± 0.006	0.057 ± 0.007	0.062 ± 0.004	0.066 ± 0.006	0.064 ± 0.003	D; 0.0146
	NE	0.087 ± 0.009	0.067 ± 0.005	0.070 ± 0.005	0.074 ± 0.007	0.079 ± 0.003	0.075 ± 0.003 *	N; 0.0044
	Diet mean ^2^	0.082 ± 0.005 ^A^	0.062 ± 0.004 ^B^	0.063 ± 0.005 ^B^	0.068 ± 0.004 ^AB^	0.073 ± 0.004 ^AB^		D*N; 0.9844
Serum Glucose (mg/dL)	PBS	118.3 ± 27.2	280.2 ± 41.3	252.5 ± 50.8	340.7 ± 41.6	162.7 ± 9.7	230.9 ± 23.6	D; 0.0003
	NE	101.0 ± 11.0	179.5 ± 49.7	114.5 ± 40.0	194.7 ± 22.2	104.0 ± 13.8	138.7 ± 15.4 *	N; 0.0002
	Diet mean ^2^	109.7 ± 14.0 ^C^	229.9 ± 35.4 ^AB^	183.5 ± 39.7 ^ABC^	267.7 ± 35.2 ^A^	133.4 ± 13.6 ^BC^		D*N; 0.3867
Serum Insulin (ng/mL)	PBS	0.20 ± 0.02	2.12 ± 0.86	0.99 ± 0.23	1.19 ± 0.51	1.12 ± 0.38	1.12 ± 0.25	D; 0.0036
	NE	0.38 ± 0.03	1.24 ± 0.29	0.35 ± 0.17	0.91 ± 0.28	0.93 ± 0.37	0.76 ± 0.13	N; 0.2604
	Diet mean ^2^	0.30 ± 0.04 ^B^	1.62 ± 0.40 ^A^	0.62 ± 0.16 ^AB^	1.03 ± 0.25 ^A^	1.01 ± 0.25 ^AB^		D*N; 0.2493
Glucose: Insulin ratio	PBS	6.07 ± 1.19	2.65 ± 1.55	3.22 ± 1.19	3.94 ± 1.52	2.10 ± 1.07	3.60 ± 0.62	D; 0.1321
	NE	2.68 ± 0.35	1.46 ± 0.17	3.34 ± 1.67	2.69 ± 0.74	2.10 ± 0.86	2.45 ± 0.31	N; 0.3044
	Diet mean ^2^	4.13 ± 0.84	1.97 ± 0.64	3.29 ± 0.69	3.22 ± 0.74	2.10 ± 0.61		D*N; 0.7640

Data are presented as means ± SEM, *n* = 7–8 for each diet group For each variable, diets were compared among each other (after combining PBS and NE data within each diet), and assigned different superscripts A, B, AB, ABC, C, or D) if they were significantly different from each other at *P* < 0.05 by Tukey’s multiple comparison test. For each variable, if a diet has a common superscript with another diet, it means they are not significantly different from each other. Only diets without a common superscript are significantly different from each other. Asterisks (*) indicate significant differences caused by NE; *P* < 0.05. ^1^ Mice were injected with norepinephrine (NE; 2 mg/kg body weight) or phosphate-buffered saline (PBS; 2 mL/kg body weight) as a vehicle after 8-h fasting at the end of the experimental period, and euthanized after another 4-h fasting. ^2^ Overall mean of each diet group. ^3^ Overall mean of PBS-treated mice or NE-treated mice.^4^ D, Diet effect; N, NE effect; D*N, Interaction effect. TRT, treatment (PBS or NE injection). TAG, triacylglycerol. NEFA, non-esterified fatty acid. CON, control; SHB, shea butter; OO, olive oil; SFO, safflower; SBO, soybean oil.

## Supplementary Materials

The following are available online at http://www.mdpi.com/2072-6643/10/2/256/s1, Figure S1: Ucp1 expression in different adipose tissue depots Mice were fed control diet containing 10% kcal fat for 12 weeks. Gene expression was determined by RT-PCR. Data are presented as means ± SEM (*n* = 4 for each tissue). Different superscript letters (A and B) indicate significant differences among groups at *P* < 0.05 by Tukey’s multiple comparison test. Table S1: Composition of the experimental diets. Table S2: The primer sequences used for quantitative Real-time PCR. Table S3: Expression of genes involved in thermogenesis in BAT. Table S4: Expression of genes involved in thermogenesis in subcutaneous WAT. Table S5: Expression of genes involved in thermogenesis in epididymal WAT. Table S6: Expression of genes involved in lipid metabolism in the liver. Table S7: Expression of genes involved in lipid metabolism in hamstring muscle. Table S8: Expression of neuropeptides and β-adrenergic receptors in hypothalamus.
