# Supplementary material for: Effects of Diets Differing in Composition of 18-C Fatty Acids on Adipose Tissue Thermogenic Gene Expression in Mice Fed High-Fat Diets
Source: Nutrients. 2018 Feb 23;10(2):256. doi: 10.3390/nu10020256 (PMC5852832; doi:10.3390/nu10020256)
Supplement: Supplementary file 1 [file nutrients-10-00256-s001.zip › C18FA in vivo NE_Supplementary Tables_revised.docx]

**Supplementary Tables**

**Table S1. Composition of the experimental diets^1^**

|  | Control  (10% Kcal Fat) | | High-fat  (45% Kcal Fat) | |
| --- | --- | --- | --- | --- |
|  | g | Kcal | g | Kcal |
| Casein | 200 | 800 | 200 | 800 |
| L-cystine | 3 | 12 | 3 | 12 |
| Sucrose | 172.8 | 691 | 172.8 | 691 |
| Cornstarch | 452.2 | 1808.8 | 72.8 | 291 |
| Dyetrose | 75 | 300 | 100 | 400 |
| SHB, OO, SFO, or SBO^2^ | 20 | 180 | 90 | 810 |
| Lard | 25 | 225 | 112.5 | 1012.5 |
| t-Butylhydroquinone | 0.004 | 0 | 0.018 | 0 |
| Cellulose | 50 | 0 | 50 | 0 |
| Mineral Mix^3^ | 35 | 0 | 35 | 0 |
| Vitamin Mix^4^ | 10 | 40 | 10 | 40 |
| Choline Bitartrate | 2 | 0 | 2 | 0 |
| Total | 1045 | 4057 | 848.1 | 4057 |
| kcal/ g diet | 3.69 | | 4.64 | |

^1^Resource: Dyets, Inc., Bethlehem, PA, USA.

^2^SHB, shea butter; OO, olive oil; SFO, safflower oil; SBO, soybean oil

^3^Thirty five grams of mineral mix (Research Diets, Inc., New Brunswick, NJ, USA, S10026) provides 1.0g sodium, 1.6g chloride, 0.5g magnesium, 0.33g sulfur, 59mg manganese, 45mg iron, 29mg zinc, 6mg copper, 2mg chromium, 1.6mg molybdenum, 0.16mg selenium, 0.9mg fluoride, 0.2 mg, iodine and 3.99g sucrose.

^4^Ten grams of vitamin mix (Research Diets, Inc., V10001) provides 4000 IU vitamin A, 1000 IU vitamin D3, 50 IU vitamin E, 30 mg niacin, 16 mg pantothenic acid, 7 mg vitamin B6, 6 mg vitamin B1, 6 mg vitamin B2, 2 mg folic acid, 0.5 mg menadione, 0.2 mg biotin, 10 μg vitamin B12 and 9.78 g sucrose.

**Table S2.** **The primer sequences used for quantitative Real-time PCR^1^**

| Gene | Function | Forward Primer | Reverse Primer |
| --- | --- | --- | --- |
| *18S* | Endogenous control | ATC CCT GAG AAG TTC CAG CA | CCT CTT GGT GAG GTC GAT GT |
| *Ucp1* | Thermogenesis | GGG CCC TTG TAA ACA ACA AA | GTC GGT CCT TCC TTG GTG TA |
| *Prdm16* | Thermogenesis | GGC TCA AGG AGG AGG AGA GA | AGG TCC GGG TCA GGT TCA TA |
| *Pgc1a* | Thermogenesis | GTC CTT CCT CCA TGC CTG AC | GTG TGG TTT GCT GCA TGG TT |
| *Tfam* | Thermogenesis | GAG CGT GCT AAA AGC ACT GG | GAC AAG ACT GAT AGA CGA GGG G |
| *Cpt1a* | Fatty acid oxidation | GGA CTC CGC TCG CTC ATT | GAG ATC GAT GCC ATC AGG GG |
| *Cpt1b* | Fatty acid oxidation | AAG TTC AGA GAC GAA CGC CC | CAG GCC TCT TCT TCC ACC AG |
| *Cpt2* | Fatty acid oxidation | GCT TTC CAA CCC GAT CTC CT | TGT GAG CGG AAG ATC CCA AC |
| *Ppara* | Transcription factor promoting fatty acid oxidation | CCC TGA ACA TCG AGT GTC GA | AAT AGT TCG CCG AAA GAA GCC |
| *Ppard* | Transcription factor promoting fatty acid oxidation | TCC ATC GTC AAC AAA GAC GGG | ACT TGG GCT CAA TGA TGT CAC |
| *Pparg* | Transcription factor promoting lipid accumulation | TTG ACC CAG AGC ATG GTG C | GAA GTT GGT GGG CCA GAA TG |
| *Npy* | Orexigenic neuropeptide | TGG CCA GAT ACT ACT CCG CT | TCT TCA AGC CTT GTT CTG GGG |
| *Agrp* | Orexigenic neuropeptide | CCC TGT CCT AGA CCT TCC AGA | GTG CCT CCC ATT GTG TTG TCT |
| *Pomc* | Anorexigenic neuropeptide | CAA CCT GCT GGC TTG CAT C | CGT ACT TCC GGG GGT TTT CA |
| *Lepr* | Leptin signaling | CAG AAT GAC GCA GGG CTG TA | AGA GTG TCC GTT CTC TTT TGG A |
| *Adrb1* | β-adrenergic signaling | CTA CAA CGA CCC CAA GTG CT | ACG TAG AAG GAG ACG ACG GA |
| *Adrb2* | β-adrenergic signaling | TAC ACA GGG GAG CCA AAC AC | TCA CAA AGC CTT CCA TGC CT |
| *Adrb3* | β-adrenergic signaling | AGT TTG AGA GCA GGA ATC CAG G | CCA AGA TGG TGC TTA GAG AGC C |
| *Gatm* | Creatine biosynthesis | TTT GCA CAG AGA AGC CAG GTT A | TGT GCA TTG GAT TGG GGT CT |
| *Serca2b* | Ca^2+^-ATPase | TTT GCC GCT CAT TTT CCA GAT CA | ACA CAC TCT TTA CCG GGT TGT T |

^1^*18S*, 18S ribosomal RNA; *Ucp1*, Uncoupling protein 1; *Prdm16*, PR domain containing 16; *Pgc1a*, Peroxisome proliferator-activated receptor gamma coactivator 1-alpha; *Tfam*, Mitochondrial transcription factor A; *Cpt1a*, Carnitine palmitoyl-transferase 1A; *Cpt1b*, Carnitine palmitoyl-transferase 1B; *Cpt2*, Carnitine palmitoyl-transferase 2; *Ppara,* Peroxisome proliferator-activated receptor α; *Ppard,* Peroxisome proliferator-activated receptor δ; *Pparg,* Peroxisome proliferator-activated receptor γ; *Npy*, Neuropeptide Y; *Agrp*, Agouti-related peptide; *Pomc*, Proopiomelanocortin; *Lepr*, Leptin receptor; *Adrb1*, Beta-1 adrenergic receptor; *Adrb2*, Beta-2 adrenergic receptor; *Adrb3*, Beta-3 adrenergic receptor; *Gatm*, Glycine amidinotransferase, mitochondrial; *Serca2b*, sarco/endoplasmic reticulum Ca^2+^-ATPase 2b.

**Table S3.** **Expression of genes involved in thermogenesis in BAT**

|  | TRT^1^ |  | 10% Fat |  | 45% Fat | | | |  | *P*-value^4^ |
| --- | --- | --- | --- | --- | --- | --- | --- | --- | --- | --- |
|  |  |  | CON |  | SHB | OO | SFO | SBO | TRT mean^3^ |  |
| *Tfam* | PBS | 1.00±0.13 | | | 1.18±0.16 | 1.16±0.35 | 1.70±0.67 | 0.90±0.09 | 1.19±0.15 | D; .3645  N; .2831  D*N; .8852 |
|  | NE | 1.42±0.34 | | | 1.00±0.11 | 1.55±0.40 | 0.95±0.33 | 0.94±0.12 | 1.37±0.14 |  |
|  | Diet mean^2^ | 1.21±0.19 | | | 1.09±0.10 | 1.35±0.26 | 1.83±0.35 | 0.92±0.07 |  |  |
| *Adrb1* | PBS | 1.00±0.25 | | | 2.16±0.63 | 1.61±0.51 | 2.28±0.74 | 1.13±0.32 | 1.64±0.24 | D; .0158  N; .6536  D*N; .8581 |
|  | NE | 1.12±0.11 | | | 1.70±0.37 | 1.22±0.38 | 2.12±0.36 | 0.80±0.09 | 1.40±0.16 |  |
|  | Diet mean^2^ | 1.06±0.13^AB^ | | | 1.93±0.35^AB^ | 1.42±0.30^AB^ | 2.20±0.38^A^ | 0.97±0.16^B^ |  |  |
| *Adrb2* | PBS | 1.00±0.29 | | | 0.97±0.46 | 0.50±0.12 | 0.69±0.16 | 0.50±0.09 | 0.73±0.12 | D; .0926  N; .0028  D*N; .2197 |
|  | NE | 0.49±0.16 | | | 0.53±0.03 | 0.38±0.08 | 0.19±0.05 | 0.40±0.07 | 0.40±0.04* |  |
|  | Diet mean^2^ | 0.75±0.18 | | | 0.75±0.23 | 0.44±0.07 | 0.44±0.12 | 0.45±0.06 |  |  |
| *Adrb3* | PBS | 1.00±0.05 | | | 2.60±0.45 | 1.16±0.38 | 1.12±0.46 | 2.18±0.45 | 1.61±0.22 | D; .0571  N; .0187  D*N; .1022 |
|  | NE | 0.91±0.29 | | | 1.38±0.37 | 0.67±0.20 | 1.33±0.26 | 0.66±0.17* | 0.99±0.13* |  |
|  | Diet mean^2^ | 0.96±0.14^AB^ | | | 1.99±0.36^A^ | 0.92±0.22^B^ | 1.22±0.25^AB^ | 1.42±0.36^AB^ |  |  |

Data are presented as means ± SEM, n = 8 for each diet group; n = 4 each for PBS and NE.

For each gene, diets were compared among each other (after combining PBS and NE data within each diet), and assigned different superscripts A, B, or AB) if they were significantly different from each other at *P* < 0.05 by Tukey’s multiple comparison test. For each gene, if a diet has a common superscript with another diet, it means they are not significantly different from each other. Only diets without a common superscript are significantly different from each other.

^1^Mice were injected with norepinephrine (NE; 2mg/kg body weight) or phosphate-buffered saline (PBS; 2mL/kg body weight) as a vehicle after 8-hour fasting at the end of the experimental period, and euthanized after another 4-hour fast. Asterisks (*) indicate significant differences caused by NE; *P* < 0.05.

^2^Overall mean of each diet group.

^3^Overall mean of PBS-treated mice or NE-treated mice.

^4^D, Diet effect; N, NE effect; D*N, Interaction effect.

TRT, treatment (PBS or NE injection). CON, control; SHB, shea butter; OO, olive oil; SFO, safflower; SBO, soybean oil.

**Table S4.** **Expression of genes involved in thermogenesis in subcutaneous WAT**

|  | TRT^1^ |  | 10% Fat |  | 45% Fat | | | |  | *P*-value^4^ |
| --- | --- | --- | --- | --- | --- | --- | --- | --- | --- | --- |
|  |  |  | CON |  | SHB | OO | SFO | SBO | TRT mean^3^ |  |
| *Tfam* | PBS | 1.00±0.16 | | | 1.54±0.29 | 1.13±0.17 | 0.71±0.22 | 1.30±0.33 | 1.14±0.12 | D; .0037  N; .1590  D*N; .5674 |
|  | NE | 0.78±0.12 | | | 1.80±0.17 | 1.59±0.15 | 0.92±0.13 | 1.85±0.59 | 1.39±0.16 |  |
|  | Diet mean^2^ | 0.89±0.10^BC^ | | | 1.67±0.16^A^ | 1.36±0.14^ABC^ | 0.81±0.12^C^ | 1.57±0.33^AB^ |  |  |
| *Adrb1* | PBS | 1.00±0.39 | | | 3.20±1.14 | 4.06±1.84 | 1.96±0.73 | 3.38±0.75 | 2.72±0.50 | D; .3094  N; .6586  D*N; .4348 |
|  | NE | 2.01±0.66 | | | 7.12±4.70 | 0.94±0.22 | 2.27±0.79 | 2.35±0.64 | 2.94±0.99 |  |
|  | Diet mean^2^ | 1.51±0.40 | | | 5.16±2.36 | 2.50±1.04 | 2.12±0.50 | 2.87±0.50 |  |  |
| *Adrb2* | PBS | 1.00±0.23 | | | 0.39±0.11 | 0.41±0.06 | 0.48±0.10 | 0.48±0.08 | 0.55±0.07 | D; .0991  N; .4997  D*N; .6491 |
|  | NE | 0.98±0.30 | | | 0.40±0.03 | 0.48±0.16 | 0.37±0.15 | 0.61±0.14 | 0.57±0.09 |  |
|  | Diet mean^2^ | 0.99±0.18 | | | 0.40±0.05 | 0.44±0.08 | 0.42±0.09 | 0.54±0.08 |  |  |
| *Adrb3* | PBS | 1.00±0.28 | | | 0.88±0.19 | 0.63±0.21 | 0.64±0.20 | 0.68±0.21 | 0.76±0.09 | D; .6837  N; .0012  D*N; .9539 |
|  | NE | 0.21±0.05 | | | 0.36±0.05 | 0.30±0.13 | 0.34±0.15 | 0.22±0.05 | 0.29±0.04* |  |
|  | Diet mean^2^ | 0.61±0.20 | | | 0.62±0.13 | 0.46±0.13 | 0.49±0.13 | 0.45±0.13 |  |  |
| *Gatm* | PBS | 1.00±0.45 | | | 1.45±0.32 | 0.83±0.14 | 1.67±0.88 | 1.16±0.12 | 1.17±0.15 | D; .1889  N; .1402  D*N; .1909 |
|  | NE | 1.33±0.67 | | | 1.41±0.63 | 0.97±0.62 | 0.16±0.10 | 0.70±0.13 | 0.91±0.23 |  |
|  | Diet mean^2^ | 1.16±0.38 | | | 1.43±0.33 | 0.90±0.29 | 0.66±0.40 | 0.93±0.12 |  |  |
| *Serca2b* | PBS | 1.00±0.13 | | | 0.90±0.26 | 0.49±0.11 | 1.10±0.45 | 0.99±0.19 | 0.88±0.09 | D; .0281  N; .2571  D*N; .3167 |
|  | NE | 0.92±0.21 | | | 1.09±0.21 | 0.42±0.13 | 0.42±0.10 | 0.77±0.14 | 0.73±0.09 |  |
|  | Diet mean^2^ | 0.96±0.11^AB^ | | | 1.00±0.16^A^ | 0.46±0.08^B^ | 0.65±0.19^AB^ | 0.88±0.12^AB^ |  |  |

Data are presented as means ± SEM, n = 8 for each diet group; n = 4 each for PBS and NE.

For each gene, diets were compared among each other (after combining PBS and NE data within each diet), and assigned different superscripts A, B, C, AB, BC, or ABC) if they were significantly different from each other at *P* < 0.05 by Tukey’s multiple comparison test. For each gene, if a diet has a common superscript with another diet, it means they are not significantly different from each other. Only diets without a common superscript are significantly different from each other.

^1^Mice were injected with norepinephrine (NE; 2mg/kg body weight) or phosphate-buffered saline (PBS; 2mL/kg body weight) as a vehicle after 8-hour fasting at the end of the experimental period, and euthanized after another 4-hour fast. Asterisks (*) indicate significant differences caused by NE; *P* < 0.05.

^2^Overall mean of each diet group.

^3^Overall mean of PBS-treated mice or NE-treated mice.

^4^D, Diet effect; N, NE effect; D*N, Interaction effect.

TRT, treatment (PBS or NE injection). CON, control; SHB, shea butter; OO, olive oil; SFO, safflower; SBO, soybean oil.

**Table S5.** **Expression of genes involved in thermogenesis in epididymal WAT**

|  | TRT^1^ |  | 10% Fat |  | 45% Fat | | | |  | *P*-value^4^ |
| --- | --- | --- | --- | --- | --- | --- | --- | --- | --- | --- |
|  |  |  | CON |  | SHB | OO | SFO | SBO | TRT mean^3^ |  |
| *Tfam* | PBS | 1.00±0.27 | | | 1.43±0.05 | 2.46±0.39 | 2.03±0.39 | 1.78±0.13 | 1.74±0.16 | D; .0012  N; .2622  D*N; .6077 |
|  | NE | 1.36±0.22 | | | 1.90±0.28 | 2.46±0.33 | 2.05±0.32 | 1.71±0.31 | 1.89±0.14 |  |
|  | Diet mean^2^ | 1.18±0.17^B^ | | | 1.67±0.16^AB^ | 2.46±0.24^A^ | 2.04±0.23^A^ | 1.74±0.15^AB^ |  |  |
| *Adrb1* | PBS | 1.00±0.10 | | | 0.91±0.08 | 2.63±0.43 | 2.87±1.18 | 1.82±0.60 | 1.85±0.31 | D; .0010  N; .3781  D*N; .7068 |
|  | NE | 0.68±0.11 | | | 1.41±0.85 | 1.67±0.24 | 3.56±0.99 | 1.41±0.47 | 1.75±0.34 |  |
|  | Diet mean^2^ | 0.84±0.09^C^ | | | 1.16±0.41^BC^ | 2.15±0.29^AB^ | 3.22±0.72^A^ | 1.62±0.36^ABC^ |  |  |
| *Adrb2* | PBS | 1.00±0.25 | | | 0.09±0.01 | 0.26±0.05 | 0.55±0.20 | 0.40±0.14 | 0.46±0.10 | D; <.0001  N; .4351  D*N; .7806 |
|  | NE | 0.88±0.16 | | | 0.11±0.03 | 0.40±0.06 | 0.45±0.13 | 0.45±0.07 | 0.46±0.07 |  |
|  | Diet mean^2^ | 0.94±0.14^A^ | | | 0.10±0.02^C^ | 0.33±0.04^B^ | 0.50±0.11^AB^ | 0.42±0.08^B^ |  |  |
| *Adrb3* | PBS | 1.00±0.52 | | | 0.16±0.03 | 0.43±0.07 | 0.54±0.18 | 0.39±0.20 | 0.51±0.12 | D; .0115  N; .2402  D*N; .5301 |
|  | NE | 0.26±0.05 | | | 0.13±0.05 | 0.61±0.14 | 0.33±0.06 | 0.27±0.11 | 0.32±0.05 |  |
|  | Diet mean^2^ | 0.63±0.28^A^ | | | 0.15±0.03^B^ | 0.52±0.08^A^ | 0.44±0.10^AB^ | 0.33±0.11^AB^ |  |  |
| *Gatm* | PBS | 1.00±0.23 | | | 2.67±0.97 | 1.58±0.18 | 1.61±0.62 | 2.26±0.63 | 1.82±0.27 | D; .3930  N; .6620  D*N; .2182 |
|  | NE | 0.97±0.11 | | | 1.16±0.20 | 1.73±0.45 | 3.59±2.02 | 1.10±0.13 | 1.71±0.43 |  |
|  | Diet mean^2^ | 0.98±0.12 | | | 1.91±0.54 | 1.66±0.23 | 2.60±1.21 | 1.68±0.37 |  |  |
| *Serca2b* | PBS | 1.00±0.17 | | | 1.23±0.29 | 0.98±0.12 | 1.22±0.78 | 1.34±0.16 | 1.15±0.12 | D; .7201  N; .4115  D*N; .8570 |
|  | NE | 1.48±0.17 | | | 1.24±0.22 | 1.00±0.19 | 1.50±0.39 | 1.29±0.25 | 1.30±0.11 |  |
|  | Diet mean^2^ | 1.24±0.14 | | | 1.24±0.17 | 0.99±0.10 | 1.36±0.37 | 1.31±0.14 |  |  |

Data are presented as means ± SEM, n = 8 for each diet group; n = 4 each for PBS and NE.

For each gene, diets were compared among each other (after combining PBS and NE data within each diet), and assigned different superscripts A, B, C, AB, BC, or ABC) if they were significantly different from each other at *P* < 0.05 by Tukey’s multiple comparison test. For each gene, if a diet has a common superscript with another diet, it means they are not significantly different from each other. Only diets without a common superscript are significantly different from each other.

^1^Mice were injected with norepinephrine (NE; 2mg/kg body weight) or phosphate-buffered saline (PBS; 2mL/kg body weight) as a vehicle after 8-hour fasting at the end of the experimental period, and euthanized after another 4-hour fast. Asterisks (*) indicate significant differences caused by NE; *P* < 0.05.

^2^Overall mean of each diet group.

^3^Overall mean of PBS-treated mice or NE-treated mice.

^4^D, Diet effect; N, NE effect; D*N, Interaction effect.

TRT, treatment (PBS or NE injection). CON, control; SHB, shea butter; OO, olive oil; SFO, safflower; SBO, soybean oil.

**Table S6.** **Expression of genes involved in lipid metabolism in the liver**

|  | TRT^1^ |  | 10% Fat |  | 45% Fat | | | |  | *P*-value^4^ |
| --- | --- | --- | --- | --- | --- | --- | --- | --- | --- | --- |
|  |  |  | CON |  | SHB | OO | SFO | SBO | TRT mean^3^ |  |
| *Cpt1a* | PBS | 1.00±0.15 | | | 1.76±0.27 | 0.86±0.04 | 0.75±0.11 | 1.16±0.15 | 1.11±0.10 | D; <.0001  N; .0014  D*N; .3902 |
|  | NE | 1.49±0.18 | | | 2.12±0.09 | 1.75±0.37 | 0.89±0.17 | 1.40±0.14 | 1.53±0.13* |  |
|  | Diet mean^2^ | 1.24±0.14^BC^ | | | 1.94±0.15^A^ | 1.31±0.24^B^ | 0.82±0.10^C^ | 1.28±0.11^B^ |  |  |
| *Cpt1b* | PBS | 1.00±0.60 | | | 0.69±0.28 | 0.51±0.07 | 0.34±0.08 | 0.46±0.12 | 0.60±0.13 | D; .1463  N; <.0001  D*N; .8161 |
|  | NE | 2.64±0.94* | | | 1.86±0.14* | 1.24±0.31 | 0.71±0.07 | 1.30±0.25* | 1.55±0.24* |  |
|  | Diet mean^2^ | 1.82±0.60 | | | 1.27±0.26 | 0.87±0.20 | 0.53±0.09 | 0.88±0.20 |  |  |
| *Cpt2* | PBS | 1.00±0.16 | | | 1.76±0.31 | 1.01±0.30 | 0.63±0.23 | 1.70±0.27 | 1.25±0.14 | D; <.0001  N; .5232  D*N; .3722 |
|  | NE | 1.57±0.27 | | | 1.70±0.22 | 0.98±0.17 | 0.37±0.05 | 2.24±0.43 | 1.37±0.18 |  |
|  | Diet mean^2^ | 1.29±0.18^AB^ | | | 1.73±0.18^A^ | 0.99±0.16^B^ | 0.48±0.11^C^ | 1.97±0.25^A^ |  |  |
| *Ppara* | PBS | 1.00±0.16 | | | 2.75±0.55 | 1.78±0.20 | 0.81±0.12 | 1.60±0.14 | 1.59±0.19 | D; <.0001  N; .6882  D*N; .3077 |
|  | NE | 0.94±0.15 | | | 2.49±0.06 | 2.15±0.36 | 0.94±0.14 | 1.06±0.16 | 1.52±0.17 |  |
|  | Diet mean^2^ | 0.97±0.10^C^ | | | 2.62±0.26^A^ | 1.97±0.20^AB^ | 0.88±0.09^C^ | 1.33±0.14^BC^ |  |  |
| *Ppard* | PBS | 1.00±0.07 | | | 2.33±0.31 | 1.46±0.21 | 0.80±0.13 | 1.63±0.06 | 1.44±0.14 | D; <.0001  N; .1157  D*N; .1621 |
|  | NE | 1.51±0.23 | | | 2.33±0.39 | 2.11±0.40 | 0.63±0.08 | 2.08±0.20 | 1.73±0.17 |  |
|  | Diet mean^2^ | 1.25±0.15^B^ | | | 2.33±0.23^A^ | 1.78±0.24^AB^ | 0.71±0.08^C^ | 1.86±0.13^A^ |  |  |
| *Pparg* | PBS | 1.00±0.63 | | | 14.97±6.04 | 2.57±1.07 | 5.91±2.17 | 11.11±5.61 | 7.11±1.95 | D; <.0001  N; .4138  D*N; .7329 |
|  | NE | 1.30±0.35 | | | 10.07±1.42 | 1.60±0.30 | 6.86±1.23 | 9.93±3.23 | 5.95±1.11 |  |
|  | Diet mean^2^ | 1.15±0.34^C^ | | | 12.52±3.02^A^ | 2.09±0.55^BC^ | 6.38±1.17^AB^ | 10.52±3.01^AB^ |  |  |
| *Adrb1* | PBS | 1.00±0.19 | | | 1.68±0.29 | 1.03±0.13 | 0.77±0.09 | 1.67±0.47 | 1.23±0.14 | D; .0037  N; .1056  D*N; .7239 |
|  | NE | 0.70±0.08 | | | 1.38±0.43 | 1.32±0.44 | 0.61±0.05 | 1.12±0.10 | 1.03±0.13 |  |
|  | Diet mean^2^ | 0.85±0.11^AB^ | | | 1.53±0.24^A^ | 1.18±0.22^AB^ | 0.69±0.05^B^ | 1.39±0.25^A^ |  |  |
| *Adrb2* | PBS | 1.00±0.20 | | | 1.73±0.47 | 1.15±0.26 | 0.73±0.17 | 1.72±0.31 | 1.27±0.15 | D; < .0001  N; .0983  D*N; .4050 |
|  | NE | 1.62±0.18 | | | 1.92±0.44 | 1.64±0.16 | 0.56±0.07 | 2.69±1.02 | 1.69±0.26 |  |
|  | Diet mean^2^ | 1.31±0.17^A^ | | | 1.83±0.30^A^ | 1.39±0.17^A^ | 0.65±0.09^B^ | 2.20±0.52^A^ |  |  |
| *Adrb3* | PBS | 1.00±0.24 | | | 3.13±0.57 | 2.98±0.84 | 1.12±0.20 | 2.80±0.53 | 2.21±0.30 | D; <.0001  N; .0060  D*N; .9523 |
|  | NE | 0.68±0.17 | | | 1.47±0.11 | 2.08±0.57 | 0.75±0.14 | 1.88±0.47 | 1.37±0.19* |  |
|  | Diet mean^2^ | 0.84±0.15^B^ | | | 2.30±0.41^A^ | 2.53±0.50^A^ | 0.94±0.13^B^ | 2.34±0.37^A^ |  |  |

Data are presented as means ± SEM, n = 8 for each diet group; n = 4 each for PBS and NE.

For each gene, diets were compared among each other (after combining PBS and NE data within each diet), and assigned different superscripts A, B, C, AB, or BC) if they were significantly different from each other at *P* < 0.05 by Tukey’s multiple comparison test. For each gene, if a diet has a common superscript with another diet, it means they are not significantly different from each other. Only diets without a common superscript are significantly different from each other.

^1^Mice were injected with norepinephrine (NE; 2mg/kg body weight) or phosphate-buffered saline (PBS; 2mL/kg body weight) as a vehicle after 8-hour fasting at the end of the experimental period, and euthanized after another 4-hour fast. Asterisks (*) indicate significant differences caused by NE; *P* < 0.05.

^2^Overall mean of each diet group.

^3^Overall mean of PBS-treated mice or NE-treated mice.

^4^D, Diet effect; N, NE effect; D*N, Interaction effect.

TRT, treatment (PBS or NE injection). CON, control; SHB, shea butter; OO, olive oil; SFO, safflower; SBO, soybean oil.

**Table S7.** **Expression of genes involved in lipid metabolism in hamstring muscle**

|  | TRT^1^ |  | 10% Fat |  | 45% Fat | | | |  | *P*-value^4^ |
| --- | --- | --- | --- | --- | --- | --- | --- | --- | --- | --- |
|  |  |  | CON |  | SHB | OO | SFO | SBO | TRT mean^3^ |  |
| *Cpt1a* | PBS | 1.00±0.32 | | | 3.27±0.95 | 2.56±0.41 | 2.23±0.34 | 1.08±0.38 | 2.03±0.29 | D; .0056  N; .0767  D*N; .3134 |
|  | NE | 1.91±0.72 | | | 3.68±0.64 | 2.11±0.42 | 2.48±0.26 | 2.00±0.23* | 2.44±0.25 |  |
|  | Diet mean^2^ | 1.46±0.40^B^ | | | 3.47±0.54^A^ | 2.33±0.28^AB^ | 2.35±0.20^AB^ | 1.54±0.27^B^ |  |  |
| *Cpt1b* | PBS | 1.00±0.16 | | | 2.70±0.22 | 2.46±0.19 | 4.57±1.16 | 1.80±0.12 | 2.50±0.35 | D; < .0001  N; .9869  D*N; .0368 |
|  | NE | 1.32±0.10 | | | 2.40±0.23 | 3.38±0.74 | 2.50±0.33* | 1.96±0.16 | 2.31±0.22 |  |
|  | Diet mean^2^ | 1.16±0.11^C^ | | | 2.55±0.16^AB^ | 2.92±0.40^AB^ | 3.53±0.68^A^ | 1.88±0.10^B^ |  |  |
| *Cpt2* | PBS | 1.00±0.10 | | | 1.38±0.27 | 0.98±0.21 | 0.70±0.15 | 1.46±0.30 | 1.13±0.11 | D; .0017  N; .6353  D*N; .9554 |
|  | NE | 1.10±0.13 | | | 1.13±0.17 | 0.93±0.12 | 0.65±0.12 | 1.29±0.06 | 1.02±0.07 |  |
|  | Diet mean^2^ | 1.05±0.08^AB^ | | | 1.26±0.16^A^ | 0.95±0.11^AB^ | 0.67±0.09^B^ | 1.37±0.14^A^ |  |  |
| *Ppara* | PBS | 1.00±0.20 | | | 4.07±0.67 | 3.77±1.11 | 4.47±1.58 | 2.33±0.25 | 3.13±0.47 | D; .0008  N; .0648  D*N; .0646 |
|  | NE | 2.46±0.36* | | | 6.55±1.96 | 2.40±0.24 | 3.39±0.85 | 4.64±1.14 | 3.89±0.57 |  |
|  | Diet mean^2^ | 1.73±0.33^B^ | | | 5.31±1.07^A^ | 3.08±0.59^AB^ | 3.95±0.85^A^ | 3.49±0.70^A^ |  |  |
| *Ppard* | PBS | 1.00±0.23 | | | 3.77±0.80 | 3.27±0.65 | 2.01±0.46 | 1.90±0.14 | 2.39±0.31 | D; .0003  N; .0003  D*N; .2038 |
|  | NE | 2.98±0.57* | | | 5.90±0.85 | 3.85±0.73 | 2.82±0.27 | 3.08±0.57 | 3.72±0.36* |  |
|  | Diet mean^2^ | 1.99±0.47^C^ | | | 4.84±0.67^A^ | 3.56±0.47^AB^ | 2.42±0.29^BC^ | 2.49±0.35^BC^ |  |  |
| *Pparg* | PBS | 1.00±0.50 | | | 9.54±3.05 | 3.95±2.61 | 4.15±1.62 | 2.66±1.38 | 4.26±1.04 | D; .0010  N; .0758  D*N; .9245 |
|  | NE | 0.66±0.44 | | | 4.31±0.92 | 2.19±0.65 | 1.69±0.73 | 0.82±0.32 | 1.93±0.40 |  |
|  | Diet mean^2^ | 0.83±0.32^B^ | | | 6.92±1.78^A^ | 3.07±1.29^AB^ | 2.92±0.94^AB^ | 1.74±0.74^B^ |  |  |
| *Adrb1* | PBS | 1.00±0.47 | | | 4.71±0.83 | 2.70±0.97 | 4.78±1.83 | 2.09±0.70 | 3.06±0.54 | D; .0015  N; .5095  D*N; .8918 |
|  | NE | 1.48±0.58 | | | 4.23±1.33 | 2.52±0.52 | 2.71±0.74 | 1.15±0.21 | 2.42±0.39 |  |
|  | Diet mean^2^ | 1.24±0.36^C^ | | | 4.47±0.73^A^ | 2.61±0.51^ABC^ | 3.74±1.00^AB^ | 1.62±0.38^BC^ |  |  |
| *Adrb2* | PBS | 1.00±0.09 | | | 1.82±0.46 | 1.00±0.11 | 2.48±1.11 | 2.80±0.91 | 1.82±0.32 | D; .1238  N; .3365  D*N; .2223 |
|  | NE | 2.64±0.57* | | | 2.28±0.66 | 1.15±0.05 | 2.12±1.02 | 1.98±0.39 | 2.03±0.27 |  |
|  | Diet mean^2^ | 1.82±0.41 | | | 2.05±0.38 | 1.07±0.06 | 2.30±0.70 | 2.39±0.48 |  |  |
| *Adrb3* | PBS | 1.00±0.48 | | | 4.49±1.49 | 1.84±1.45 | 1.92±0.63 | 5.49±4.79 | 2.95±1.02 | D; .1711  N; .0811  D*N; .3841 |
|  | NE | 1.40±1.11 | | | 1.68±0.60 | 1.32±0.33 | 0.30±0.12* | 1.39±0.71 | 1.22±0.29 |  |
|  | Diet mean^2^ | 1.20±0.56 | | | 3.09±0.91 | 1.58±0.69 | 1.11±0.43 | 3.44±2.37 |  |  |

Data are presented as means ± SEM, n = 8 for each diet group; n = 4 each for PBS and NE.

For each gene, diets were compared among each other (after combining PBS and NE data within each diet), and assigned different superscripts A, B, C, AB, or BC) if they were significantly different from each other at *P* < 0.05 by Tukey’s multiple comparison test. For each gene, if a diet has a common superscript with another diet, it means they are not significantly different from each other. Only diets without a common superscript are significantly different from each other.

^1^Mice were injected with norepinephrine (NE; 2mg/kg body weight) or phosphate-buffered saline (PBS; 2mL/kg body weight) as a vehicle after 8-hour fasting at the end of the experimental period, and euthanized after another 4-hour fast. Asterisks (*) indicate significant differences caused by NE; *P* < 0.05.

^2^Overall mean of each diet group.

^3^Overall mean of PBS-treated mice or NE-treated mice.

^4^D, Diet effect; N, NE effect; D*N, Interaction effect.

**Table S8.** **Expression of neuropeptides and β-adrenergic receptors in hypothalamus**

|  | TRT^1^ |  | 10% Fat |  | 45% Fat | | | |  | *P*-value^4^ |
| --- | --- | --- | --- | --- | --- | --- | --- | --- | --- | --- |
|  |  |  | CON |  | SHB | OO | SFO | SBO | TRT mean^3^ |  |
| *Npy* | PBS | 1.00±0.17 | | | 0.57±0.09 | 0.88±0.18 | 0.63±0.18 | 0.61±0.10 | 0.74±0.07 | D; .2584  N; .2376  D*N; .3851 |
|  | NE | 0.87±0.10 | | | 0.70±0.25 | 0.75±0.10 | 1.03±0.18 | 0.89±0.13 | 0.85±0.07 |  |
|  | Diet mean^2^ | 0.94±0.09 | | | 0.63±0.13 | 0.82±0.10 | 0.83±0.14 | 0.75±0.09 |  |  |
| *Agrp* | PBS | 1.00±0.13 | | | 0.63±0.19 | 1.24±0.27 | 0.87±0.13 | 0.90±0.04 | 0.93±0.08 | D; .1844  N; .2821  D*N; .2086 |
|  | NE | 0.95±0.11 | | | 1.03±0.24 | 0.93±0.17 | 0.84±0.10 | 1.58±0.35 | 1.07±0.10 |  |
|  | Diet mean^2^ | 0.97±0.08 | | | 0.83±0.16 | 1.08±0.16 | 0.85±0.07 | 1.24±0.21 |  |  |
| *Pomc* | PBS | 1.00±0.12 | | | 1.40±0.64 | 1.01±0.38 | 1.33±0.72 | 1.64±0.52 | 1.28±0.21 | D; .3427  N; .7490  D*N; .6512 |
|  | NE | 1.00±0.15 | | | 1.56±0.44 | 0.66±0.11 | 0.57±0.07 | 2.22±0.56 | 1.20±0.19 |  |
|  | Diet mean^2^ | 1.00±0.09 | | | 1.48±0.36 | 0.84±0.19 | 0.95±0.36 | 1.93±0.37 |  |  |
| *Lepr* | PBS | 1.00±0.54 | | | 1.67±1.42 | 0.50±0.15 | 0.31±0.20 | 0.87±0.75 | 0.87±0.32 | D; .9608  N; .1132  D*N; .4320 |
|  | NE | 0.20±0.10 | | | 0.27±0.16 | 0.22±0.06 | 0.31±0.12 | 0.37±0.14 | 0.28±0.05 |  |
|  | Diet mean^2^ | 0.60±0.30 | | | 0.97±0.71 | 0.36±0.09 | 0.31±0.11 | 0.62±0.36 |  |  |
| *Adrb1* | PBS | 1.00±0.22 | | | 1.12±0.27 | 1.07±0.29 | 0.60±0.13 | 1.12±0.15 | 0.98±0.10 | D; .0369  N; .0829  D*N; .6751 |
|  | NE | 0.82±0.26 | | | 0.75±0.24 | 0.62±0.08 | 0.48±0.05 | 1.23±0.19 | 0.78±0.09 |  |
|  | Diet mean^2^ | 0.91±0.16^AB^ | | | 0.94±0.18^AB^ | 0.85±0.16^AB^ | 0.54±0.07^B^ | 1.18±0.11^A^ |  |  |
| *Adrb2* | PBS | 1.00±0.13 | | | 1.14±0.24 | 1.34±0.24 | 0.83±0.16 | 1.62±0.20 | 1.19±0.10 | D; .0001  N; .7753  D*N; .2080 |
|  | NE | 1.09±0.10 | | | 1.46±0.21 | 0.91±0.12 | 0.75±0.03 | 1.88±0.19 | 1.22±0.11 |  |
|  | Diet mean^2^ | 1.04±0.08^BC^ | | | 1.30±0.16^AB^ | 1.13±0.15^BC^ | 0.79±0.08^C^ | 1.75±0.14^A^ |  |  |
| *Adrb3* | PBS | 1.00±0.13 | | | 0.94±0.31 | 1.11±0.56 | 0.66±0.14 | 1.33±0.25 | 1.03±0.14 | D; .0074  N; .3380  D*N; .8187 |
|  | NE | 1.19±0.07 | | | 1.15±0.23 | 0.82±0.10 | 0.53±0.02 | 1.89±0.34 | 1.12±0.13 |  |
|  | Diet mean^2^ | 1.09±0.08^AB^ | | | 1.05±0.18^AB^ | 0.96±0.27^AB^ | 0.56±0.09^B^ | 1.61±0.22^A^ |  |  |

Data are presented as means ± SEM, n = 8 for each diet group; n = 4 each for PBS and NE.

For each gene, diets were compared among each other (after combining PBS and NE data within each diet), and assigned different superscripts A, B, AB, BC, or C) if they were significantly different from each other at *P* < 0.05 by Tukey’s multiple comparison test. For each gene, if a diet has a common superscript with another diet, it means they are not significantly different from each other. Only diets without a common superscript are significantly different from each other.

^1^Mice were injected with norepinephrine (NE; 2mg/kg body weight) or phosphate-buffered saline (PBS; 2mL/kg body weight) as a vehicle after 8-hour fasting at the end of the experimental period, and euthanized after another 4-hour fast. Asterisks (*) indicate significant differences caused by NE; *P* < 0.05.

^2^Overall mean of each diet group.

^3^Overall mean of PBS-treated mice or NE-treated mice.

^4^D, Diet effect; N, NE effect; D*N, Interaction effect.

TRT, treatment (PBS or NE injection). CON, control; SHB, shea butter; OO, olive oil; SFO, safflower; SBO, soybean oil.
