# Supplementary figures and images for: Effects of Diets Differing in Composition of 18-C Fatty Acids on Adipose Tissue Thermogenic Gene Expression in Mice Fed High-Fat Diets
Source: Nutrients. 2018 Feb 23;10(2):256. doi: 10.3390/nu10020256 (PMC5852832; doi:10.3390/nu10020256)

**Figure S1**

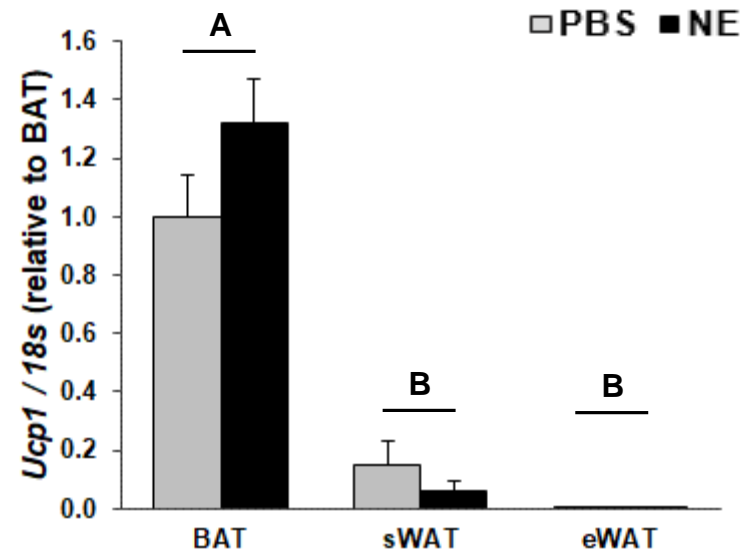

Tissue,  $P < .0001$ ; NE,  $P = .3042$ ; T\*N,  $P = .0766$

Supplement: Supplementary file 1 [file nutrients-10-00256-s001.zip › C18FA in vivo NE_Supplementary Figure.pdf]
